# Intraportal Rupture of a Hepatic Hydatid Cyst Complicated by Arterioportal Fistula and Hepatic Artery Pseudoaneurysm

**DOI:** 10.7759/cureus.117020

**Published:** 2026-09-27

**Authors:** Kawtar Khaissidi, Ismail Chaouche, Hajar Ouazzani, Amal Akammar, Nizar El Bouardi, Meriem Haloua, Badreddine Alami, Meryem Boubbou, Mustapha Maaroufi, Moulay Youssef Alaoui Lamrani

**Affiliations:** 1 Department of Radiology, Hassan II University Hospital, Fez, MAR

**Keywords:** arterioportal fistula, endovascular embolization, hepatic artery pseudoaneurysm, hepatic hydatid cyst, intraportal rupture

## Abstract

A 71-year-old woman with known hepatic hydatid disease presented with fever, abdominal pain, vomiting, and jaundice. Magnetic resonance imaging (MRI) with MR cholangiopancreatography revealed rupture of a right hepatic hydatid cyst with intraportal hydatid dissemination associated with a hepatic artery pseudoaneurysm and an arterioportal fistula. The patient rapidly developed severe acute cholangitis with multiorgan failure. Endoscopic retrograde cholangiopancreatography (ERCP) allowed extraction of the hydatid material and biliary stenting, followed by selective endovascular embolization of the hepatic artery pseudoaneurysm. Despite combined endoscopic and interventional management, the patient progressed to refractory multiorgan failure and died. The present case illustrates a rare and life-threatening vascular complication of hepatic hydatid disease, emphasizing the importance of early imaging diagnosis and multidisciplinary management. In the present case, simultaneous portal and arterial involvement was associated with an unfavorable outcome.

## Introduction

Hepatic hydatid disease is a parasitic infection endemic in many regions, most commonly affecting the liver and frequently complicated by intrabiliary rupture, which may lead to biliary obstruction, cholangitis, and secondary infection [1]. However, vascular involvement, particularly portal vein rupture with intraportal dissemination and associated arterial complications, remains exceptionally rare and poorly described. Arterioportal fistulization may further result in significant hemodynamic consequences by increasing portal venous inflow and aggravating portal hypertension. Such presentations can lead to complex biliary, vascular, and infectious sequelae requiring multidisciplinary management. We report an exceptionally rare case of hepatic hydatidosis, characterized by the simultaneous occurrence of intraportal hydatid dissemination, portal thrombosis, an arterioportal fistula, and a right branch of the proper hepatic artery pseudoaneurysm, an unusual combination of portal and arterial complications.

## Case presentation

A 71-year-old woman with a medical history of hypertension, type 2 diabetes mellitus, and right knee prosthesis, who was being followed for known hepatic hydatid disease, presented to the emergency department with acute abdominal pain, vomiting, fever, and jaundice.

Physical examination revealed marked jaundice and tenderness in the right upper quadrant and epigastrium.

Laboratory tests showed a marked inflammatory response, with a C-reactive protein level of 199 mg/L, associated with thrombocytopenia (87,000/mm³). Liver function tests demonstrated a cholestatic pattern, with a total bilirubin level of 68 mg/L (conjugated bilirubin, 51 mg/L), alkaline phosphatase of 257 IU/L, and gamma-glutamyl transferase of 289 IU/L. Transaminases were also elevated (aspartate aminotransferase (AST), 117 IU/L; alanine aminotransferase (ALT), 251 IU/L), and the prothrombin time was reduced to 39%.

Initial abdominal ultrasonography demonstrated a 68-mm hydatid cyst in the right hepatic lobe, associated with dilatation of the intrahepatic bile ducts containing intraluminal material. Magnetic resonance imaging (MRI) with MR cholangiopancreatography was subsequently performed to further characterize the biliary involvement and investigate possible associated complications.

MRI and MR cholangiopancreatography demonstrated a hydatid cyst involving hepatic segments VII and VIII, with rupture into the right portal vein and multiple daughter vesicles disseminated within the portal venous system. Early enhancement of the portal vein during the arterial phase indicated an arterioportal fistula. In addition, a contrast-enhancing outpouching arising from the right branch of the proper hepatic artery was identified, consistent with a hepatic artery pseudoaneurysm (Figure 1).

**Figure 1:**
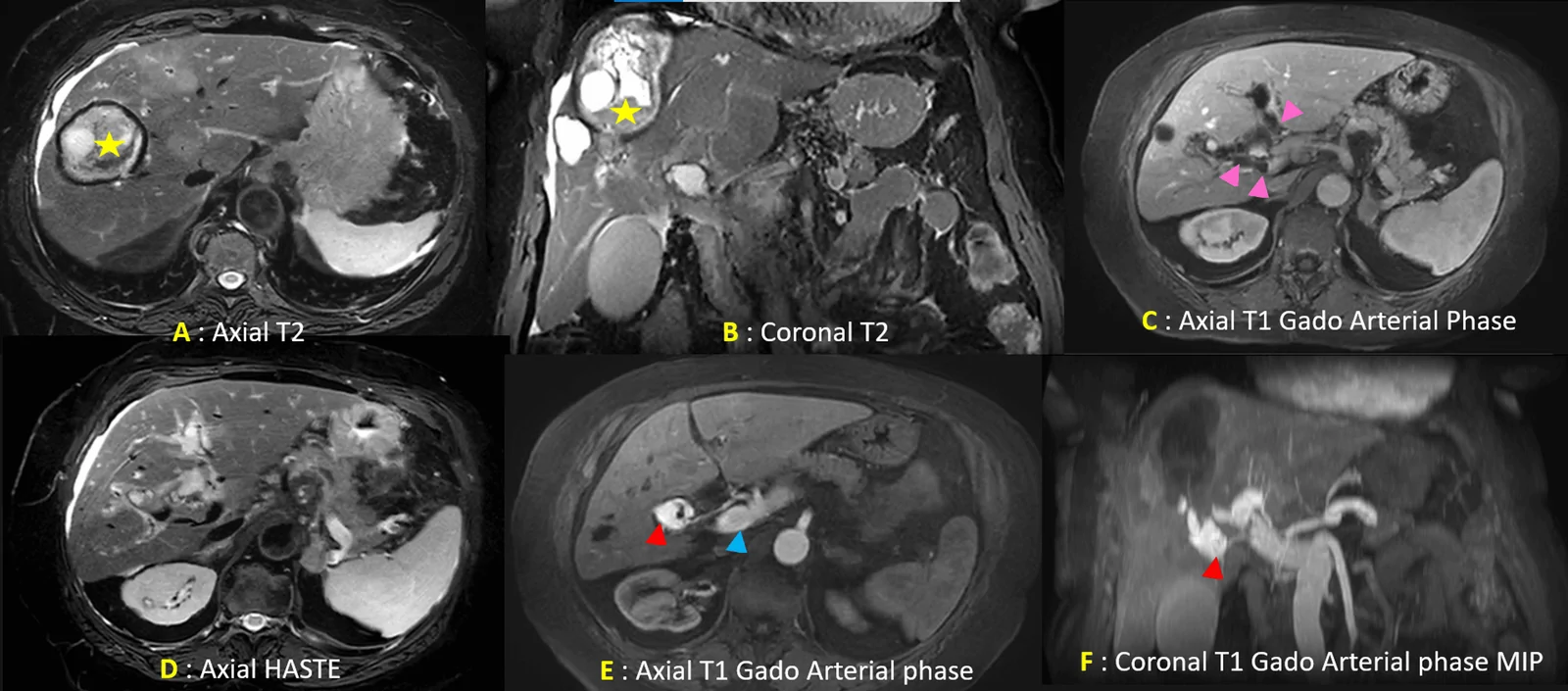
MRI findings of the complicated hepatic hydatid cyst. (A-B) Axial and coronal T2-weighted images show a hydatid cyst straddling hepatic segments VII and VIII (yellow star). (C) Axial contrast-enhanced T1-weighted image obtained during the arterial phase demonstrates multiple daughter vesicles within the right portal vein and its branches (pink arrowheads), confirming intraportal rupture of the hydatid cyst. (D) Axial HASTE image demonstrates intraportal hydatid involvement. (E) Axial contrast-enhanced T1-weighted image obtained during the arterial phase demonstrates early opacification of the portal vein, consistent with an arterioportal fistula (blue arrowhead), and a contrast-enhancing outpouching arising from the right branch of the proper hepatic artery, consistent with a pseudoaneurysm (red arrowhead). (F) Coronal contrast-enhanced T1-weighted arterial phase maximum intensity projection (MIP) image demonstrates the hepatic artery pseudoaneurysm (red arrowhead). MRI: magnetic resonance imaging; HASTE: half-Fourier acquisition single-shot turbo spin-echo; Gado: gadolinium

At admission, the patient fulfilled the grade III acute cholangitis criteria, with associated organ dysfunction including respiratory failure, hepatic dysfunction with a prothrombin time of 39%, and thrombocytopenia (87,000/µL). She was admitted to the intensive care unit (ICU) and managed with resuscitation, broad-spectrum antibiotics, and supportive therapy.

She underwent endoscopic retrograde cholangiopancreatography (ERCP), which showed dilation of the intrahepatic and common bile ducts with a distal filling defect. Several hydatid membranes, daughter vesicles, and thick debris were extracted using balloon sweeps. A 9 cm plastic stent was placed, resulting in effective biliary drainage. On follow-up computed tomography (CT), the same findings were confirmed, including hydatid material within the portal venous branches, a persistent arterioportal fistula, and a pseudoaneurysm arising from the right branch of the proper hepatic artery, along with a large-volume intraperitoneal effusion (Figure 2).

**Figure 2:**
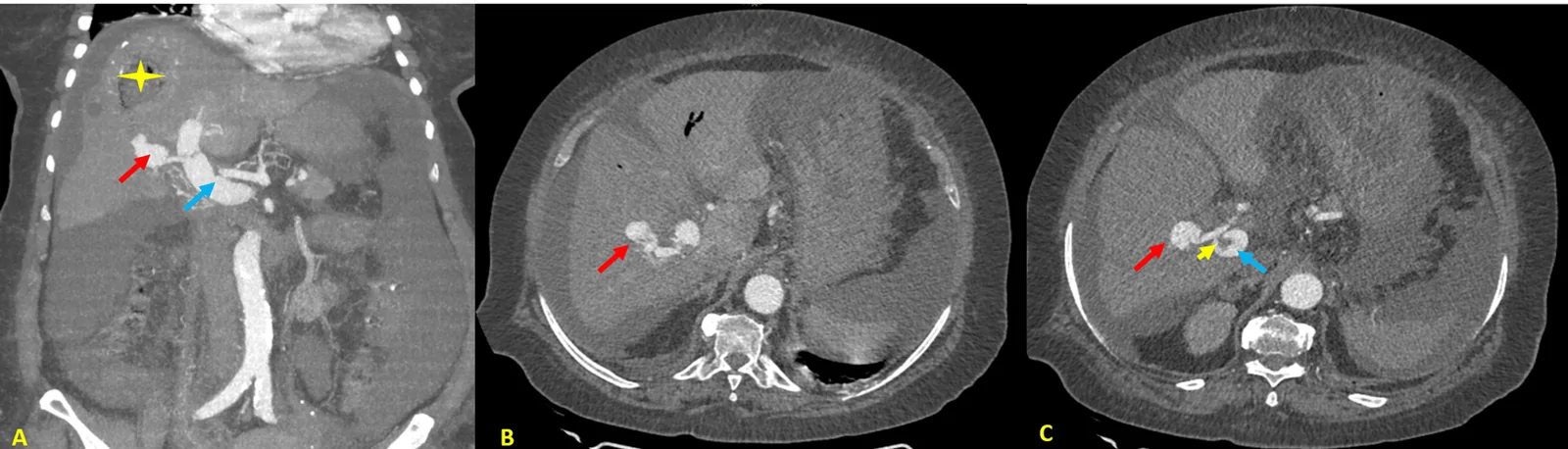
(A) Contrast-enhanced coronal CT image obtained during the arterial phase with maximum intensity projection (MIP) reconstruction demonstrates the hydatid cyst of the hepatic dome (yellow star), a contrast-enhancing pseudoaneurysm arising from the right branch of the proper hepatic artery (red arrow), and early opacification of the portal vein, consistent with an arterioportal fistula (blue arrow). (B) Contrast-enhanced axial CT image obtained during the arterial phase demonstrates the hepatic artery pseudoaneurysm (red arrow). (C) Contrast-enhanced axial CT image obtained during the arterial phase demonstrates the hepatic artery pseudoaneurysm (red arrow), early opacification of the portal vein consistent with an arterioportal fistula (blue arrow), and a focal filling defect within the portal vein consistent with partial portal thrombosis (yellow arrow). CT: computed tomography

Endovascular embolization was performed. Via right femoral access, selective catheterization of the celiac trunk and common hepatic artery revealed a large aneurysmal dilation of the right branch of the proper hepatic artery with a retrograde arterio-portal shunt causing marked dilation of the portal trunk and left portal branch. Super-selective embolization of the right branch of the proper hepatic artery proximal to the aneurysmal sac was achieved using two coils, followed by a mixture of lipiodol and biological glue, resulting in complete exclusion of the pseudoaneurysm (Figure 3).

**Figure 3:**
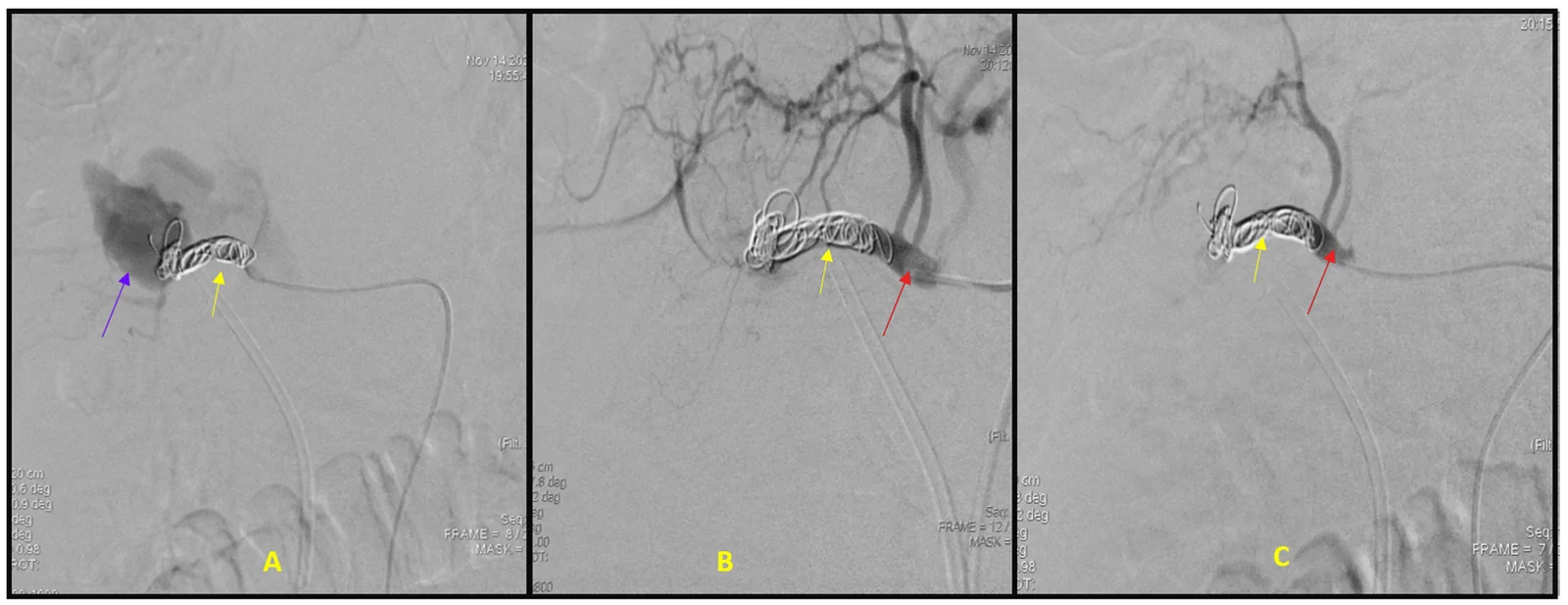
Angiographic images demonstrating selective microcatheterization of the right branch of the proper hepatic artery and endovascular embolization of the pseudoaneurysm. (A) Pre-embolization angiography demonstrates the hepatic artery pseudoaneurysm (blue arrow) and the embolization coils (yellow arrow). (B) Angiographic image during endovascular treatment demonstrates the embolization coils (yellow arrow) and the right branch of the proper hepatic artery (red arrow). (C) Post-embolization angiography demonstrates complete exclusion of the pseudoaneurysm, with the embolization coils in place (yellow arrow) and the hepatic artery indicated by the red arrow.

The clinical course was marked by the development of multiorgan failure, with respiratory distress, delirium, recurrent ascites, and multifactorial renal insufficiency. Despite successful endovascular exclusion of the right branch of the hepatic artery pseudoaneurysm and maximal supportive therapy, the patient's condition continued to deteriorate, with refractory multiorgan failure. She died in the ICU on the third day following endovascular embolization.

## Discussion

Hydatid disease of the liver remains endemic in many regions, including Morocco, where more than 80% of cases involve the liver [1]. Although usually benign, its complications can be severe, and while intrabiliary rupture is common, vascular involvement such as invasion of the portal venous system or other major vessels remains exceptionally rare [1-2]. Most series emphasize that the usual spectrum of complications includes biliary obstruction, cholangitis, compression-related portal hypertension, Budd-Chiari syndrome, and intraperitoneal rupture, but only isolated cases describe direct portal vein invasion or cavernomatosis due to hydatid cysts [3].

Intrabiliary rupture occurs in approximately 3-25% of hepatic hydatid cysts and represents the most common complication, typically presenting with obstructive jaundice or acute cholangitis [4]. MRI and MRCP play a crucial role in detecting cystobiliary communication and intraductal hydatid debris, while ERCP is central to management, allowing removal of hydatid membranes and daughter cysts, performing sphincterotomy when required, and placing a biliary stent to ensure adequate drainage [5].

Vascular complications of hepatic hydatid cysts are rare, and rupture into major or adjacent vessels has been estimated to occur in approximately 0.05% of cases [6]. Portal venous involvement has been reported in several forms, including hydatid thrombosis of the portal vein, cavernomatous transformation due to compression or invasion, and, more rarely, direct rupture of the cyst into the portal trunk or its branches [7].

Recent radiological studies have emphasized the crucial role of cross-sectional imaging in differentiating bland portal vein thrombosis from intravascular hydatid material and in identifying daughter vesicles within the portal system [8]. Our case contributes to this limited literature by demonstrating bilateral intraportal hydatid dissemination associated with portal thrombosis and portal hypertension. Previous reports have also described multivessel thrombosis in complicated hepatic hydatid disease, suggesting that embolization of hydatid material and intense local inflammatory reactions may promote extensive portal and hepatic venous thrombosis, potentially leading to liver failure [9].

In our patient, we hypothesize that portal hypertension and recurrent ascites may have amplified the hemodynamic consequences of portal obstruction and arterioportal shunting. This mechanism may have contributed to the subsequent clinical deterioration and multiorgan failure; however, a direct causal relationship cannot be established from this single case.

Vascular complications of hydatid disease involving arteries are even more exceptional. Systematic and narrative reviews of arterial hydatid cysts and individual case reports describe pseudoaneurysms of the hepatic, aortic, and other visceral arteries, usually in the context of infected or ruptured cysts eroding into the vascular wall [10].

A few reports specifically link intrabiliary hydatid disease with hepatic artery pseudoaneurysm and hemobilia, underlining the capacity of chronic inflammation and enzymatic erosion to weaken the arterial wall [11].

In our case, the exact mechanism underlying pseudoaneurysm formation could not be definitively established. We hypothesize that local inflammatory changes related to the complicated hydatid cyst may have contributed to the weakening of the adjacent arterial wall. Imaging demonstrated a pseudoaneurysm of the right branch of the proper hepatic artery associated with a retrograde arterioportal shunt and marked dilatation of the portal trunk and the left portal branch. Endovascular treatment with coil and glue embolization of the pre-aneurysmal segment was technically successful and is consistent with the current practice favoring minimally invasive embolization for visceral artery pseudoaneurysms in high-risk surgical candidates [12].

The association in a single patient of ruptured and superinfected hepatic hydatid cyst, intrabiliary rupture with severe cholangitis, intraportal hydatid dissemination arterioportal fistula, and hepatic artery pseudoaneurysm appears exceptional, even when compared with recent case series and reviews of “disastrous” complications of hepatic hydatid disease [3].

This constellation dramatically complicates management because each component carries its own risk of hemorrhage, sepsis, or liver failure. In addition, the treatment must be staged: urgent ERCP to control cholangitis, antimicrobial therapy and organ support in ICU, and delayed endovascular exclusion of the pseudoaneurysm. In the present case, despite appropriate stepwise management, including endoscopic drainage, broad-spectrum antibiotics, intensive supportive care, and successful embolization, the patient progressed to persistent multiorgan dysfunction and death. This unfavorable outcome suggests that simultaneous portal and arterial involvement may be associated with a poor prognosis, although no general prognostic conclusion can be drawn from a single case.

This case therefore underscores several key learning points: in endemic areas, hydatid disease should be considered in any patient with cholangitis, portal vein thrombosis, or unusual hepatic vascular lesions; MRI/MRCP and multiphasic CT/angiography are essential to detect complex biliary and vascular fistulas and to differentiate hydatid material from bland thrombus.

ERCP plays a central role in the acute management of hydatid-related cholangitis; early involvement of interventional radiology is crucial when vascular complications such as pseudoaneurysm or arterio-portal shunt are present.

## Conclusions

This case report aims to highlight an exceptionally rare presentation of hepatic hydatid disease combining intraportal rupture with major arterial complications, and emphasize the pivotal role of imaging in their early recognition. Cross-sectional imaging, particularly CT and MRI, is essential for identifying intraportal hydatid material and associated vascular complications, including arterioportal fistula and hepatic artery pseudoaneurysm. Early recognition of these complications is highly crucial for guiding appropriate multidisciplinary management combining endoscopic, endovascular, and intensive supportive care when required. In our case, simultaneous portal and arterial involvement was associated with an unfavorable outcome, highlighting the severity and therapeutic complexity of this rare presentation. However, no general prognostic conclusion can be drawn from a single case.
